# Back-Analyses of Landfill Instability Induced by High Water Level: Case Study of Shenzhen Landfill

**DOI:** 10.3390/ijerph13010126

**Published:** 2016-01-12

**Authors:** Ren Peng, Yujing Hou, Liangtong Zhan, Yangping Yao

**Affiliations:** 1 Department of Civil Engineering, Beihang University, No.37 Xue-Yuan Road, Beijing 100191, China; ypyao@buaa.edu.cn; 2 Institute of Geotechnical Engineering, China Institute of Water Resources and Hydropower Research, No.20 Che-Gongzhuang West Road, Beijing 100048, China; houyj@iwhr.com; 3 Institute of Geotechnical Engineering, MOE Key Laboratory of Soft Soils and Geo-environmental Engineering, Zhejiang University, No.866 Yu-Hangtang Road, Hangzhou 310058, China; zhanlt@zju.edu.cn

**Keywords:** landfill instability, high water level, back analyses

## Abstract

In June 2008, the Shenzhen landfill slope failed. This case is used as an example to study the deformation characteristics and failure mode of a slope induced by high water levels. An integrated monitoring system, including water level gauges, electronic total stations, and inclinometers, was used to monitor the slope failure process. The field measurements suggest that the landfill landslide was caused by a deep slip along the weak interface of the composite liner system at the base of the landfill. The high water level is considered to be the main factor that caused this failure. To calculate the relative interface shear displacements in the geosynthetic multilayer liner system, a series of numerical direct shear tests were carried out. Based on the numerical results, the composite lining system simplified and the centrifuge modeling technique was used to quantitatively evaluate the effect of water levels on landfill instability.

## 1. Introduction

Since millions of tons of municipal solid waste (MSW) are being dumped onto “garbage mountains” every day, disposal of this waste is an increasing concern throughout the world [1]. Further, as MSW consists of large amount of organic and vegetable wastes, the leachates from these wastes cause leachate levels to rise, which affects both the stability of the slope and the drainage of polluted water. These are new engineering geology and hydrogeology problems. For example, Hendron *et al.* [2] and Caicedo *et al.* [3] independently concluded that high pore water pressure in an excessively wet waste body were mainly responsible for the instability and failure of the Dona Juana landfill in Bogota, Colombia. Koerner and Soong [4] found that for ten large solid waste landfill failures, the triggering mechanisms were excessive liquid in the waste and buildup of pore water pressure. Four of them were due to the buildup of leachate in the waste resulting in the failure surfaces occurring above the low permeable soil or geomembrane liner at the base of the landfills. In China, a large proportion of MSW is kitchen waste, which produces a significant amount of leachate. If the discharge mechanism is clogged, or the long-term drainage is impeded, this leachate accumulates thereby causing the water level in the landfill to rise. A survey shows that in China, there are many existing landfills have high water level problems, which may seriously challenge the stability of the landfills [5]. Therefore, there is an urgent need to study the deformation characteristics and failure mode of landfills with high water levels. This study has identified the factors that are important in the prediction and evaluation of landfill stability, and for the prevention of landfill instability.

The failure mechanism and the mode of landfill instability have been studied through field investigations [6,7,8,9], physical model tests [10,11] and numerical simulations [12,13,14]. However, none of these studies used an integrated system to monitor the landfill. Such a system can monitor the surface horizontal displacement, the deep lateral displacement, and the water level.

In this paper, the Shenzhen landfill (Shenzhen Xiaping landfill) landslide case study is presented. This landfill has undergone severe deformation and failure. An integrated monitoring system, including water level gauges, electronic total stations, and inclinometers, was used to monitor the failure process. A case report documenting these field monitoring data has been published previously in Chinese [1]. The present study is a continuation of that earlier work, aiming to back-analyse the landfill instability induced by the high water level. The centrifuge modeling technique was used to quantitatively evaluate the effect of water level on the landfill instability.

## 2. Geological Setting

**Figure 1:**
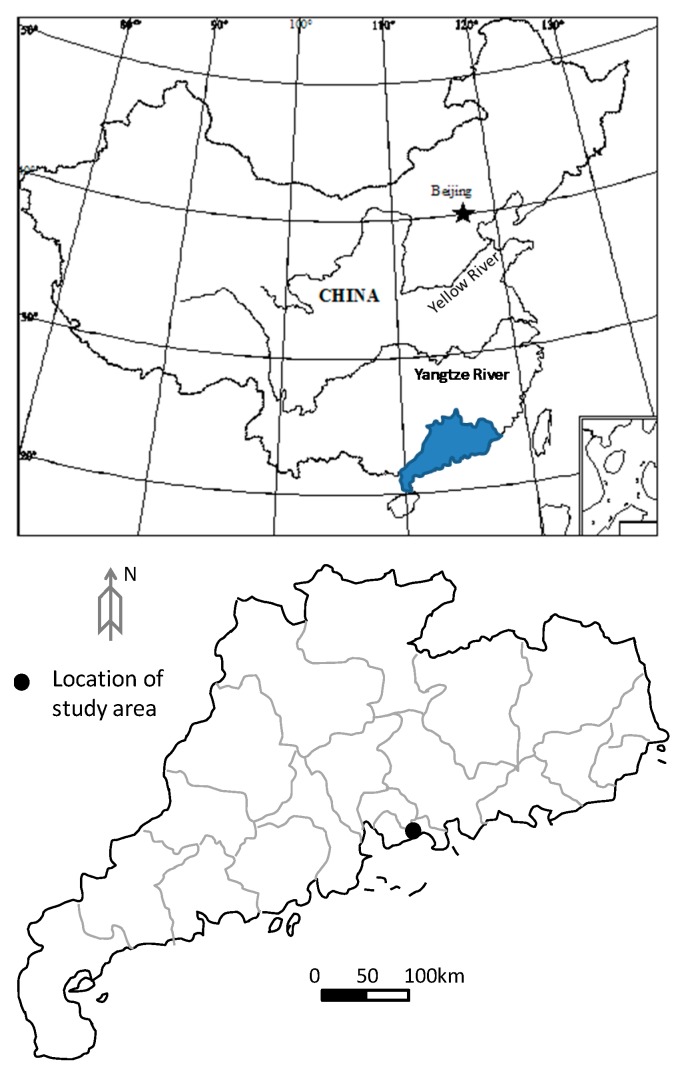
Location of the Shenzhen landfill.

**Figure 2:**
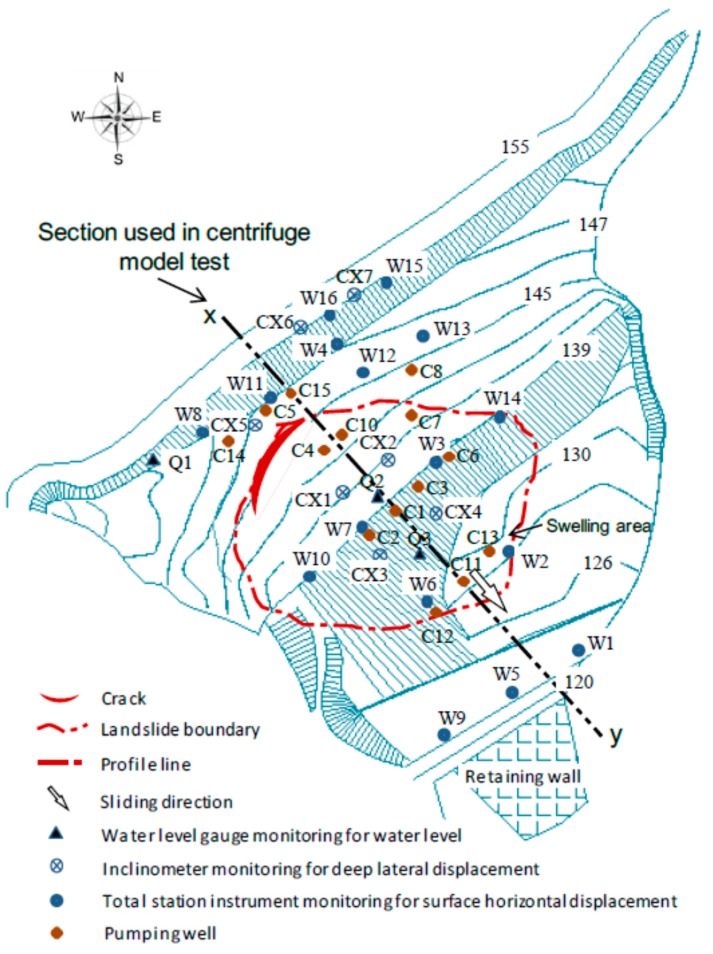
Site plan of monitoring points and pumping wells.

As shown in Figure 1, the Shenzhen landfill is located in the village of Caopu, Shenzhen City, Guangdong Province, China. It is a typical valley type landfill. The topography at the base of the landfill is in a north to south direction As shown in Figure 2, a retaining wall was built at the narrow valley end to hold the waste body. Behind the retaining wall in the north-west direction, the slope of the waste body was between 1:3.5 and 1:4. The maximum height of the waste body was about 40 m. At this landsite, the topography varies greatly. On the west side, there is a ridge that extended under the waste body, making the terrain very steep. The slope of the terrain along the base of the waste body was as steep as 24°. The bedrock mainly consists of sandstone (*i.e*., siltstone and quartz sandstone). There is no weak intercalated layer or structural plane. The ground is therefore considered a solid foundation.

**Figure 3:**
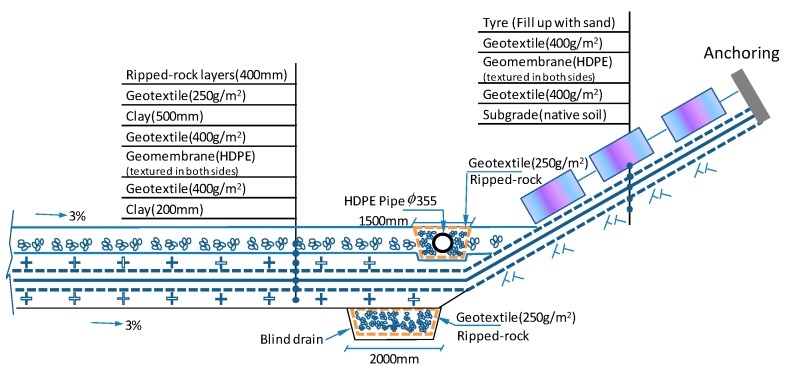
Structural drawing of the composite liner system.

The Shenzhen landfill was one of the earliest sanitary landfills equipped with a composite liner system, as shown in Figure 3. Based on research results and practical experience, in the composite liner, the shear strength is low at the high-density polyethylene (HDPE) geomembrane/geotextiles and the compacted clay/geotextiles interface [15]. Hence, these are the weak interfaces for the stability of the waste body.

In 2000, the waste started to accumulate behind the retaining wall. The main components were municipal solid waste and a small amount (<10%) of construction waste. The interim covering layer was clayey soil containing gravel, and the thickness was between 0.5 and 1.0 m. The age of the filled waste ranged between 3 and 10 years, with the oldest waste at the bottom and the youngest at the top.

**Figure 4:**
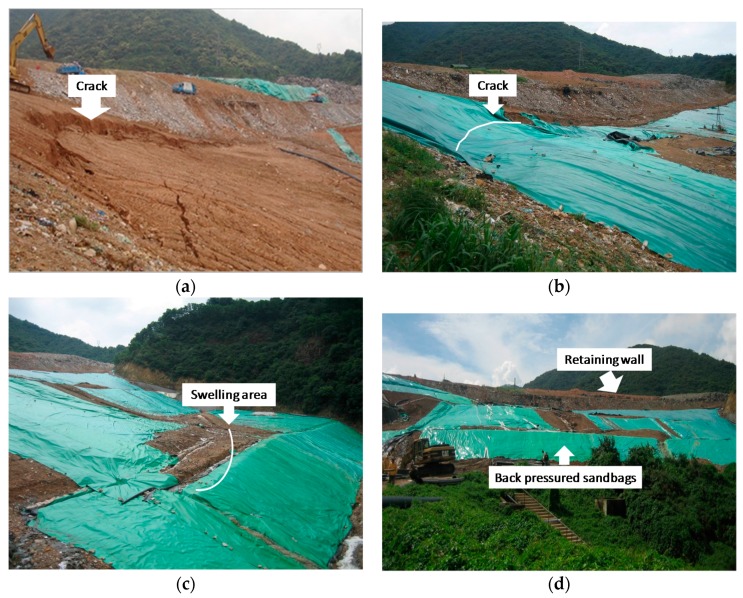
Shenzhen landfill in June 2008 (**a**) Crack at the trailing edge of waste body; (**b**) Plastic mulch paved on the waste body surface; (**c**) Swelling area; (**d**) Retaining wall.

During the heavy rainfall events in 17–18 April 2008, the waste body slid by 1~2 m. The slip area was on the west side, about one-third from the top of the waste body. During another heavy rainfall event in 6–7 June 2008, the middle part and lower part of the waste body slid by 1~4 m. As shown in Figure 4, due to the slip, a ridge of more than 3 m in height was formed. There was heave in the slip area which extended from one-third to two-thirds from the top of the waste body.

## 3. Field Monitoring and Emergency Management

According to the field observations, in early March 2008, a crack was formed on the west side near the top of the waste body. As such, that area was in a state of instability. Therefore, the local government decided to monitor the deformation of the landfill slope so that emergency actions could be carried out as necessary. The field monitoring included water level gauges for the monitoring of water levels, electronic total stations for the monitoring surface horizontal displacements, and inclinometers for the monitoring of deep horizontal displacements.

### 3.1. Monitoring of Water Level

There were a total of 10 water level gauges; three were installed in the water pipes (identified as Q1–Q3 in Figure 2). The other seven were installed in the inclinometer casing (CX1–CX7 in Figure 2). The water level was measured using a standpipe piezometer interrogated with a dipmeter.

**Figure 5:**
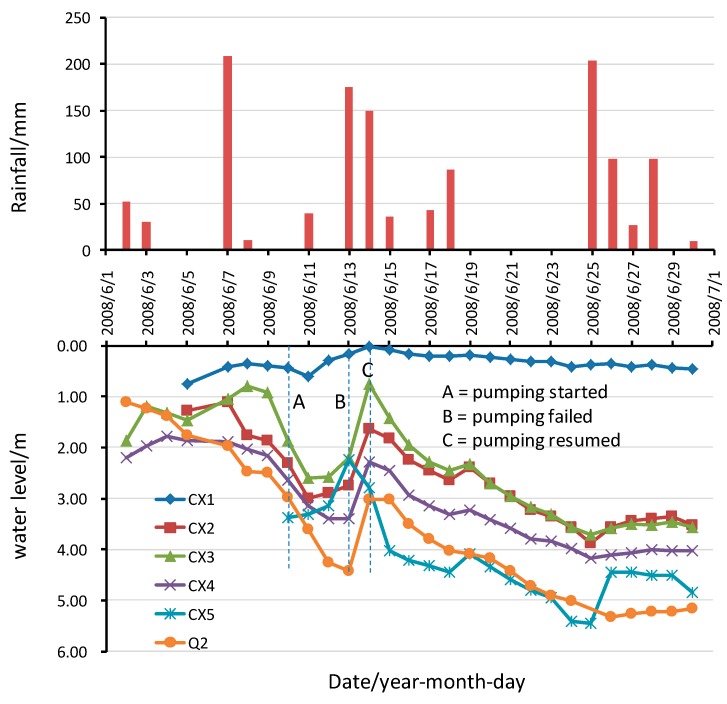
Change of water level with time and rainfall conditions.

Figure 5 shows some of the monitored water level data and rainfall data in June, 2008. Water depth was measured from the surface of the waste body which was taken as zero. Water level gauges CX2~CX5 and Q2 were within the slip area, which also had dewatering wells during the 2008 emergency. As shown in Figure 5, the water level within the waste body was very high at the beginning of June, and the water surface was 2 m below the surface of the waste body. It was observed onsite that leachate water mixture overflowed near the retaining wall, which eventually caused the waste body to slide during the 6~7 June heavy rainfall event. On 10 June, the construction of 15 wells within the waste body started, and plastic mulch was paved on the waste body surface so as to reduce infiltration of rainwater into the body. On 13 June, due to heavy rain and the failure of the pumping system, the water level again rose quickly . Subsequently, the pumping resumed and the water level fell quickly. The water levels in gauges CX2~CX5 all dropped to 3.5 m below the surface, and the water level in gauge Q2 dropped to 5.0 m below the surface. Regarding the gauge CX1, it was located in a position where there was a rise in topography. The thickness of the waste body was thin, and due to the supply of leachate and water from upstream, the water level change was not obvious.

### 3.2. Monitoring of Surface Horizontal Displacement

To capture the state of sliding of the waste body in a timely manner, total station instruments were installed so as to monitor the horizontal surface movements. A total of sixteen (W1–W16) surface displacement monitoring gauges were installed. The gauges W12–W16 were installed for the purpose of monitoring the displacements within the slip zone, which were greater after the 6–7 June heavy rainfall event.

**Figure 6:**
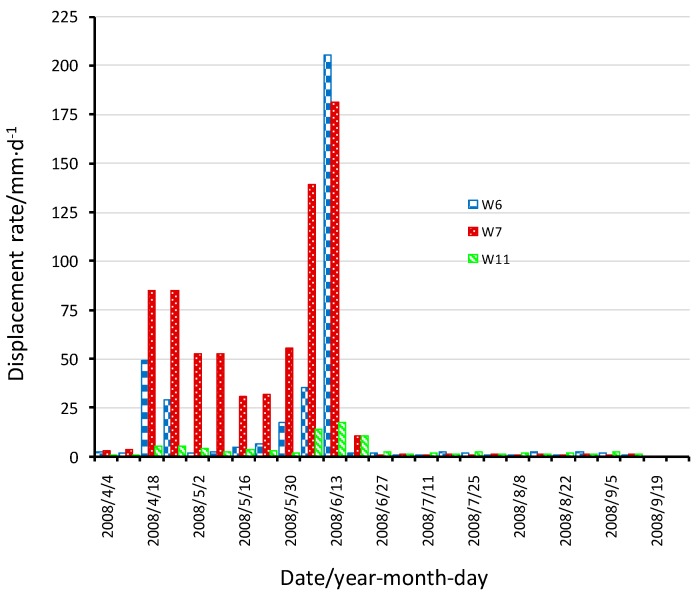
Surface horizontal displacement rates for gauges W6, W7 and W11.

The surface horizontal displacement rate in the waste body is a direct reflection of the landfill movement. As the monitoring frequency varied according to the season and the displacement rate (Table 1), Figure 6 shows the adjusted monitored surface horizontal displacement rates from March–October, 2008. Since the recordings of the W6, W7, W11 gauges respectively represent the horizontal surface displacements of the lower, middle and top part the waste body (Figure 2), only the results from these three gauges are shown in Figure 6.

**Table 1:** Horizontal displacement rate and monitoring frequency during dry and rainy seasons.

Meteorological Condition	Dry Season	Dry Season	Rainy Season	Rainy Season
Horizontal displacement rate (DR)	DR < 5 mm/d	5 mm/d > DR < 10 mm/d	5 mm/d > DR < 10 mm/d	DR > 10 mm/d
Monitoring frequency	Once a month	Once a week	Once a day	Twice a day

Considering the rainfall condition in June and the rise in water level, there were increases in the average daily displacement rates for all three gauges. In particular, the large displacement rates in 6–7 June, and 13 June correspond to the two slides. As shown in Figure 6, the gauge W7 shows that the average daily surface horizontal displacement rates at the centre of the waste body were 135 mm/d and 185 mm/d during the two slides. The average displacement rate in the centre of the waste body is generally greater than that for the rest of the body. The displacement rate of the posterior region in the waste body is also generally smaller. This shows that the gauges W6 and W7 were within the same slip plane, with the centre part of the waste body moving outward.

### 3.3. Monitoring of Deep Lateral Displacement

At the monitoring point, elevation of the bottom of the landfill and the thickness of the waste can be determined based on geological data. The boreholes could be drilled to the required depth, where the bottom of the boreholes kept a certain distance (ranged from 3 to 5 m) from the liner. Inclinometer casings were then lowered into the borehole, and subsequently these boreholes were backfilled with gravel (3 to 6 mm in grain size). The bottom of the inclinometer casing was not fixed, so both the surface horizontal displacement monitoring and the deep lateral displacement monitoring ought to be carried out simultaneously. In this way, by combining the monitored horizontal displacement at the top of the inclinometer with the monitored lateral displacement at different depths of the inclinometer, the actual displacement at different depths of the waste body can then be derived.

**Figure 7:**
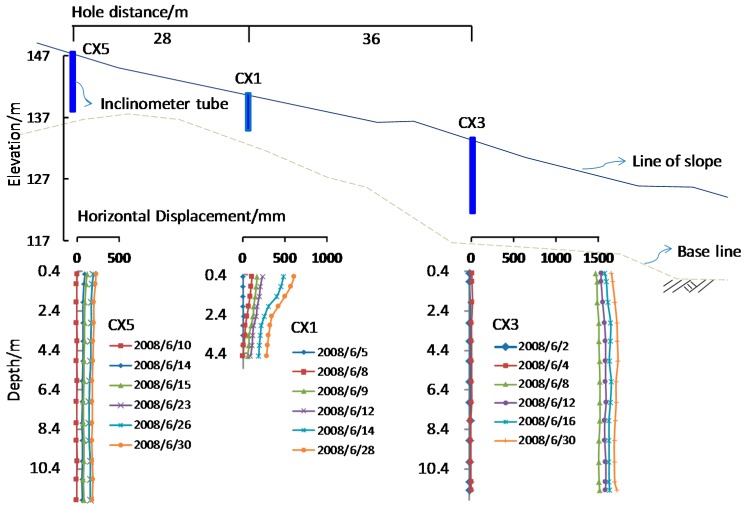
Monitored deep lateral displacements at various time.

Figure 2 shows the locations of the deep lateral displacement monitor points. Using the monitoring results of gauges CX1, CX3 and CX5, Figure 7 shows a set of typical monitoring results. These three gauges are in a line parallel to the x–y line. The gauge CX3 was located in the middle of the waste body. The gauge CX1 was located at the bulging area at the lower part of the waste body. The gauge CX5 was located near the top of the waste body. Figure 7 shows that there was very little displacement at the lower part of the waste body, while there was large displacement in the middle part. The recordings of gauge CX5 show that the displacements are consistent throughout the entire depth of the gauge, where the displacement was as large as 1.5 m on 8 June. At this location, the depth of the sliding waste body was about 12 m. A comparison of the displacement results of the three gauges show that the slip surface went through the middle-lower part of the CX1 inclined tube, but not through the CX5 tube. This result suggests that the deformation occurred at the deep composite liner system. The translational slide is the most likely failure mode.

### 3.4. Emergency Management

After the landslide, a variety of emergency measures have been carried out, which subsequently proved to be effective. After the 6–7 June heavy rainfall event, 15 pumping wells were drilled into the waste body, as shown in Figure 2. It can be seen from Figure 5 that after the start of the pumping, the water level in the wells fell. At the same time, a plastic film was laid on top of the waste body so as to reduce infiltration to the body. Further, the drains were unclogged so that the surface water in concave area could flow out. At the point where the leachate overflowed, the drainage pipe was connected to a gravel ditch so that the leachate could flow out. Finally, sandbags were used to exert a back pressure to the bulging area at the foot of the slope. After these rescue measures, it can be seen from Figure 6 that the displacement rates of the waste body slowed down significantly. In the subsequent heavy rainfall event, there was no obvious slip. These emergency measures played a key role in preventing further landslide during the 2008 rainy season.

## 4. Analysis of the Landslide Mechanisms

The use of liner systems for landfill design and construction brings some challenges. Construction of a landfill includes the placement of soil cover and waste layers up to various heights over these liners can result in application of substantial down-slope shear stresses on the liners leading to development of significant liner tension. In addition, interface strengths of the composite liner system may not be enough to resist the increased gravity forces gravity, which resulted in deep sliding along the weak interface at the bottom of landfill.

Jones and Dixon [16] stated that the design of landfills must consider the stability both within and between elements of a lining system. However, when a liner system is anchored on one side, the displacement of the geosynthetics on that side is then limited. As such, the distribution of forces within each component is complex as it is mainly due to the deformability and frictional interaction between components. Therefore, to use an analytical method to determine the tensile stress in the geomembrane, and the displacement between each interface becomes more difficult. Hence, numerical simulation is often used to simulate the composite liner interface characteristics and the tensile properties of the composite liner [17,18,19,20,21]. The explicit difference modelling code, FLAC (Fast Lagrangian Analysis of Continua in 2-Dimensions) (Itasca International Inc., Minnesota, MN, USA) [22], is the most commonly used numerical analysis technique for simulating landfill lining systems.

Based on the field monitoring data, it can be deduced that the slip is most likely to occur at the side slope. Therefore, FLAC was mainly used to study the characteristics of the composite liner system at the side slope of Shenzhen landfill. In the current study, a three-layer geosynthetic lining system has been analyzed. Beam elements attached to sub-grids on both side with interfaces have been used to represent the geosynthetic elements (Itasca 2007) [22]. A series of direct shear tests were used to simulate the behavior of the critical interface in the geosynthetic multilayer liner system. 

**Figure 8:**
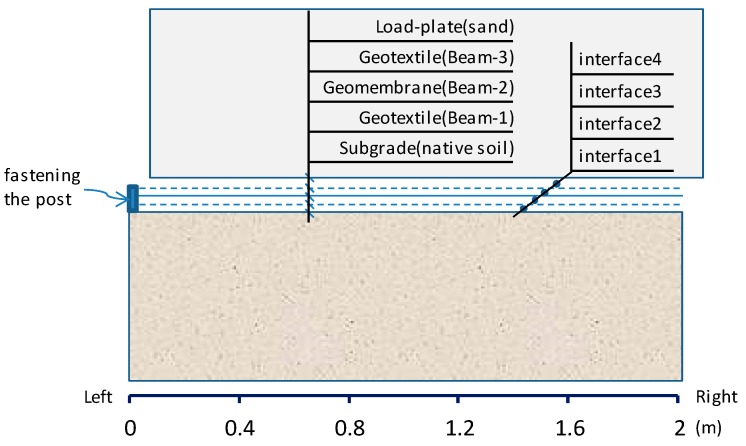
Composite liner system in direct shear test.

In the simulation model, the length has been set at 2 m. In the model, the left side of the liner system has been fixed so as to simulate the operation condition in the field where the fastening posts were anchored at the top of the slope, as shown in Figure 8.

**Figure 9:**
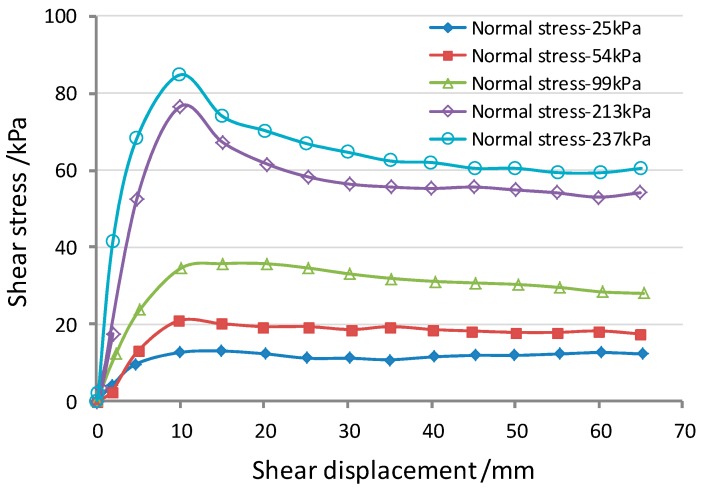
Geotextile-geomembrane friction tests (reproduced from Lin, 2009 [15]).

The beams were modelled using a linear elastic law. The tensile strength data for the textured HDPE geomembranes (GM) and the geotextiles (GT) were acquired from the laboratory tests, as shown in Table 2. The interface behavior was assured to be elastic-perfectly with a Mohr-Coulomb failure criterion. The interface parameters resulting from the shear box characterization tests are shown in Table 3. Villard *et al.* [18] describes the method on how to determine the values of these parameters. The system used, and the results obtained for the GT/GM interface, are shown in Figure 9. An elastic element with a bulk modulus of 2e5kPa, and a shear modulus of 1e5kPa was used to simulate the load-plate (sand) and subgrade (native soil).

**Table 2:** Mechanical properties of geosynthetics (modified from Lin, 2009 [15]).

Geosynthetics	Thickness (mm)	Tensile Stiffness (kN/m)
Textured HDPE geomembrane	1.5	380
Geotextile(400 g/m^2^)	4	33.3

**Table 3:** Friction parameters of the various interfaces (modified from Lin, 2009 [15]).

Interface	Cohesion (kPa)	Friction Angle (°)	Normal Stiffness (MPa/m)	Shear Stiffness (MPa/m)
Granular soil/GT	0	24	70	7
GT/GM	3	19.8	60	6
GT/Native soil	0	25	40	4

A series of interface tests conducted on a large direct shear apparatus (made by Geotest Company, Shaoxing, China), and the standard used in the test is ASTM (American Society for Testing Material) D5321. Interfaces are given the normal and shear stiffness values, and the interface shear strength.

**Figure 10:**
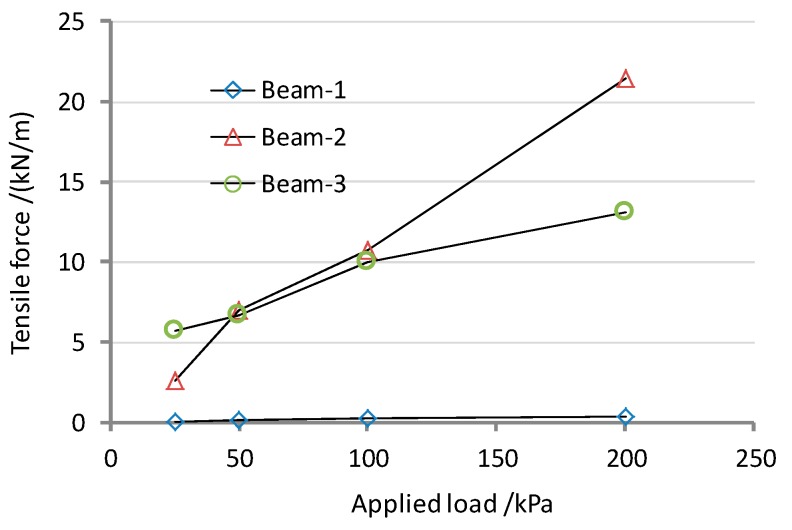
Tension in the geosynthetic, at anchorage from FLAC models.

**Figure 11:**
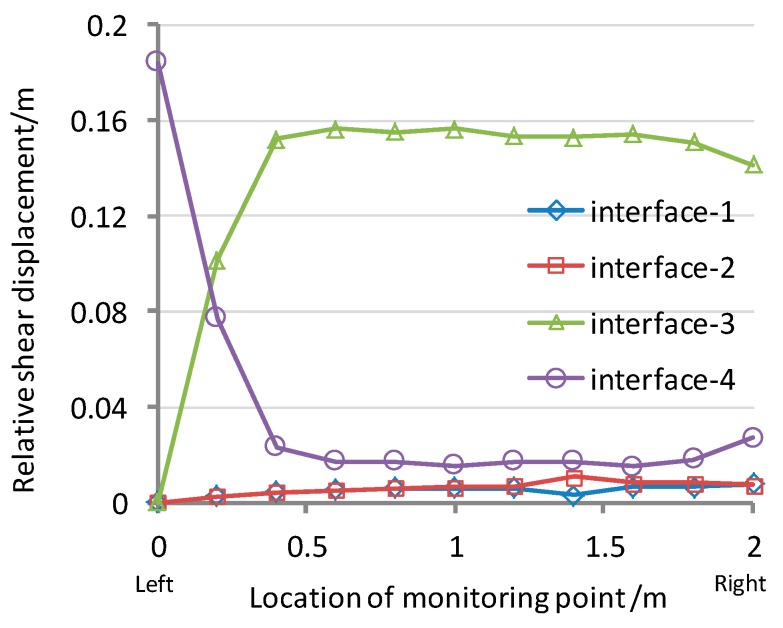
Relative interface shear displacements between different interfaces (Normal stress = 100 kPa).

In the simulated direct shear test, a constant shearing speed of 1 mm/min was used together with the normal stresses of 25, 50, 100 and 200 kPa. The tension developed at the beam anchorages in all of the numerical analysis is shown in Figure 10. It can be seen that the pulling force of the geosynthetic materials is dependent on the position. The relative shear displacements at the interfaces are shown in Figure 11. As the peak strength is minimum at the GT/GM interface, the largest relative shear displacements occurred at interface-3, and the displacements measured between the synthetic waste and the geotextile (interface-4) do not reach the post peak values. While the characteristics of interface-2 and interface-3 are similar, but since the GM tensile stiffness is relatively higher, it therefore undertakes a larger shear force coming from the upper part of the composite liner. This limits the relative displacement transferring to interface-2 and interface-1. So, there is only a small tensile stress in the geotextile under the GM, as shown in Figure 10.

According to the results of the numerical models, the trends of the tensile stresses in the geosynthetics and the relative displacements at the interface give the quantitative data. The sliding path within composite liner at the side slope of Shenzhen landfill is most likely to occur between the upper GT/GM interfaces (interface 3).

Therefore, when considering the stability of a landfill which consists of a composite liner layer, it is important to know how much stress is transferred between the interfaces. The weakest interface as well as the most likely slip surface must be identified, so that a reasonable conclusion can be drawn.

## 5. Back-Analysis of the Waste Landslide

**Figure 12:**
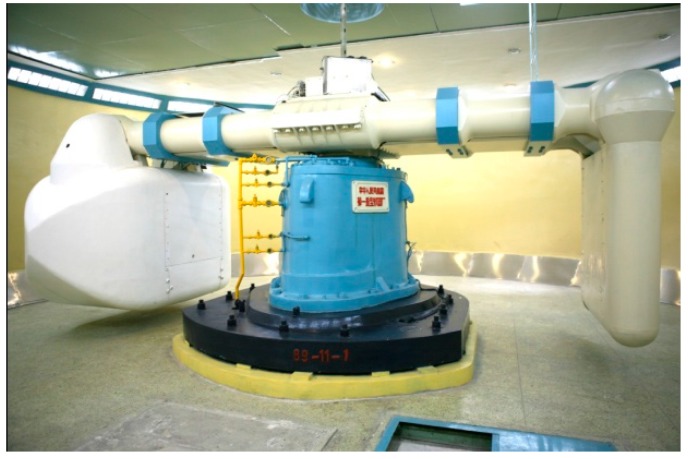
The centrifuge facility at IWHR.

To further understand the Shenzhen landfill landslide, an experiment using a physical model was carried out at an enhanced-g level. In order to generate the enhanced-g, a geotechnical centrifuge at China Institute of Water Resources and Hydropower Research (IWHR) was used. The IWHR geotechnical centrifuge apparatus has a design capacity of 450g–ton, a maximum model acceleration of 300 g and a maximum payload mass of 1500 kg, as shown in Figure 12. The centrifuge modeling technique is commonly used to examine deformations and instability of geological slopes [23]. Earlier studies on groundwater fluctuations induced slope instability in a centrifuge include Timpong *et al.* [24], Zheng and Tang [25], Knappett [26] and Yang *et al.* [27]. Previous studies by the authors were based on simple hypothetical steep slope models of inclinations of 60°–90° (*i.e*., initially unstable slopes) [28]. The main objective of this experiment is to gain a quantitative understanding between the variations in pore water pressure and their effect on the slope landfill stability. In addition, none of the earlier studies made reference to a full-scale landfill landslide.

### 5.1. Preparation of Model Slope

Fine sand, kaolin clay, and peat are the main materials used to model the MSW. The stress-strain curves, density, void ratio, shear strength, permeability coefficient, loading and unloading characteristics parameters could fit well with some of the features of the MSW (Zhu, *et al.* [11]; Peng, *et al.* [29]). In this study, the model waste was assumed isotropic, and the anisotropy effects (especially permeability anisotropy) were not considered. The moisture content of model waste was 45%. To construct the model slope, the model waste was compacted, such that the unit weight and void ratio should be equal to 9 kN/m^3^ and 1.6 respectively. Table 4 shows the main properties of the model waste.

**Table 4:** Physical properties of model waste.

Model Waste	Fill Age	Moisture Content	Void Ratio	Unit Weight	Coefficient of Compressibility	Permeability Coefficient	Shear Strength
Middle aged waste	3.6–6.6 year	45%	1.6	9 kN/m^3^	2.62 MPa^−1^	6.6 × 10^−4^ cm/s	Cohesion = 15 kPa, φ = 28°

**Figure 13:**
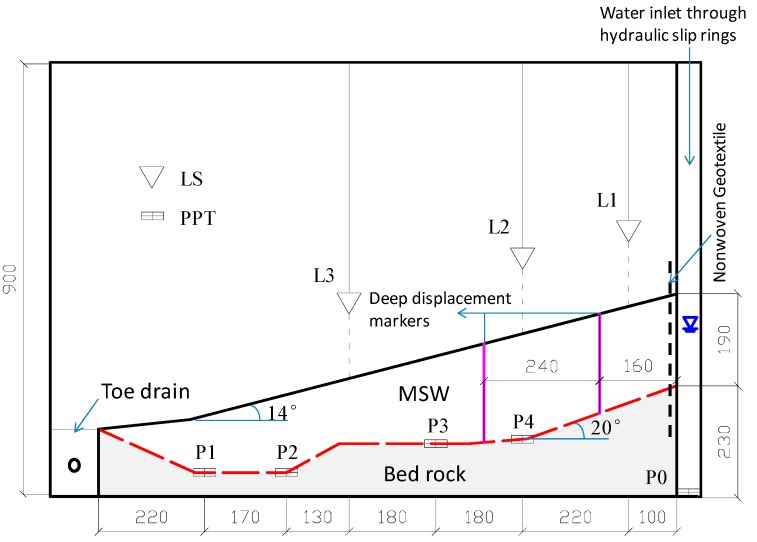
Experimental setup of model slope (Note: all dimensions are in mm).

The model was constructed in a rigid aluminum box with inner dimensions of 135 × 40 × 90 cm (length × width × height). Based on the section map along the x–y profile of the Shenzhen landfill (Figure 2), the gravity acceleration in the model was designed to be 90 g. Figure 13 shows the model slope, which corresponds to the prototype slope of approximately 108 m long.

If an acceleration of N times the Earth’s gravity (g) is applied to the centrifuge model, according to the scaling laws, the tension stiffness must be N times smaller in the model as compared to the prototype, if the interface frictional behavior is to be the same as the prototype [11]. It is impractical to recreate completely a prototype composite liner system structure in the centrifugal model test. A simplified composite liner system was therefore made for the model.

Based on the previous analysis, the weakest interface of the composite liner layer is the upper GT/GM interface. Because the tensile stiffness of GT is reduced by one order of magnitude than GM, the deformation of the GM is much smaller than that of GT. So, in this experiment, the GM which used in the field was adhered to the base of the model. A thinner GT with a thickness of 0.5 mm, a tensile strength smaller than that in the actual site, but has similar interface frictional angles (Figure 9) was anchored to the GM on the right side.

To measure the surface settlements of the slope, three laser displacement transducers (LS) were placed on the surface of the slope. To measure the pore water pressure within the slope, four pore pressure transducers (PPT) were installed at the base of the model waste body. Figure 13 shows the positions of LS and PPT. One additional PPT (P0 in Figure 13) was placed at the bottom of seepage tank.

### 5.2. Test Procedures and Results

**Figure 14:**
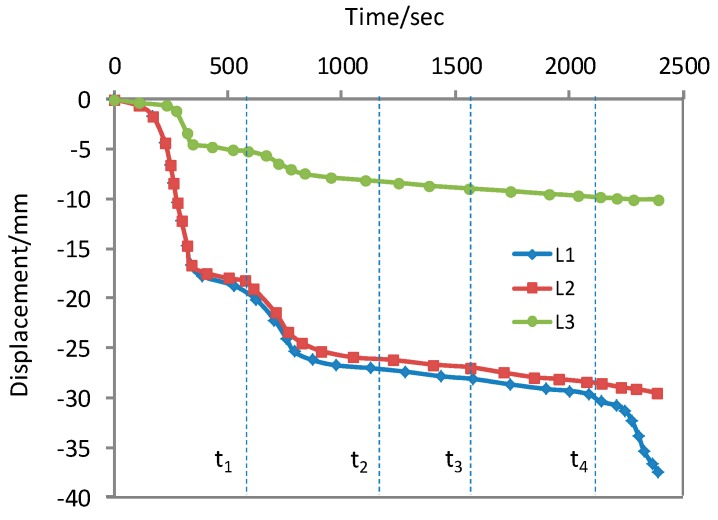
Variations of surface settlement with time in model slope.

**Figure 15:**
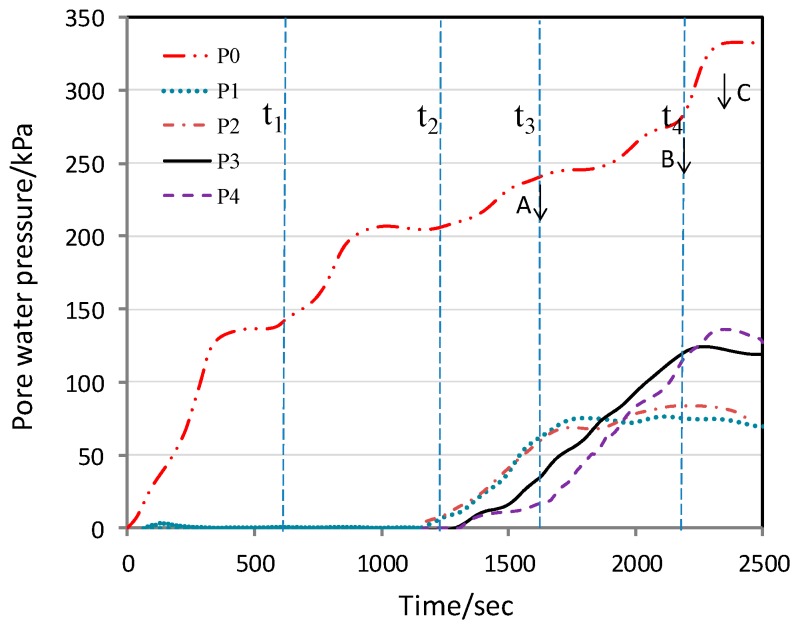
Variations of pore water pressure with time in model slope.

The centrifuge was subjected to a stepwise increase to the design acceleration of 90 g. Figure 14 and Figure 15 respectively show the monitored time histories of the surface settlements and pore water pressures during the centrifuge flight. Upon reaching the design test acceleration, the water level in the model waste body was raised by introducing water into the water supply chamber at the upslope end at time t_2_. Figure 15 clearly shows at time t_2_, the positive pore water pressure started to buildup. Between time t_2_ and t_3_, the water supply to the chamber was kept at a constant rate of 800 mL/min and the groundwater table moved upward. This period between time t_2_ and t_3_ was approximately 7 min. When the rate of water supply to the chamber increased to 2000 mL/min at time t_4_, there was a significant surface settlement as shown by the monitored displacement of L1 transducer. This phenomenon is useful to predict the occurrence of slope failure in the test model (because L1 laid above the side slope in the model, if it slips at this area, an additional settlement occurs).

**Figure 16:**
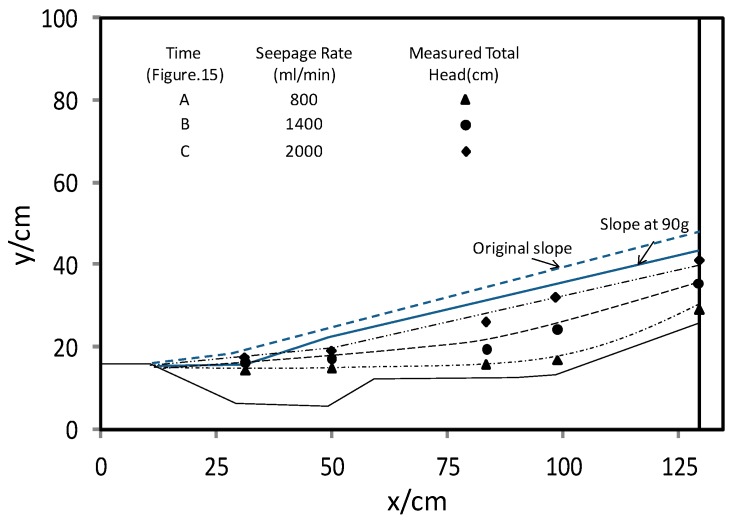
Variations of ground water level in model slope.

Back calculations were performed using the measured pore water pressure to establish the variations of groundwater table in the model slope, as shown in Figure 16. When the rate of water supply to the chamber was at 2000 mL/min, the back calculations show that the depth between the ground surface and the groundwater table was about 2.5 cm (corresponding to 2.25 m in the prototype). This result is very similar to the monitored data in the field on 6~7 June. During the model testing between time t_3_ and t_4_, flooding was observed near the toe of the slope. In the back calculations, the groundwater table appeared to be higher than the ground surface. This may be attributed to the water flowing out through the tension cracks, which resulted in flooding at the toe of the slope.

**Figure 17:**
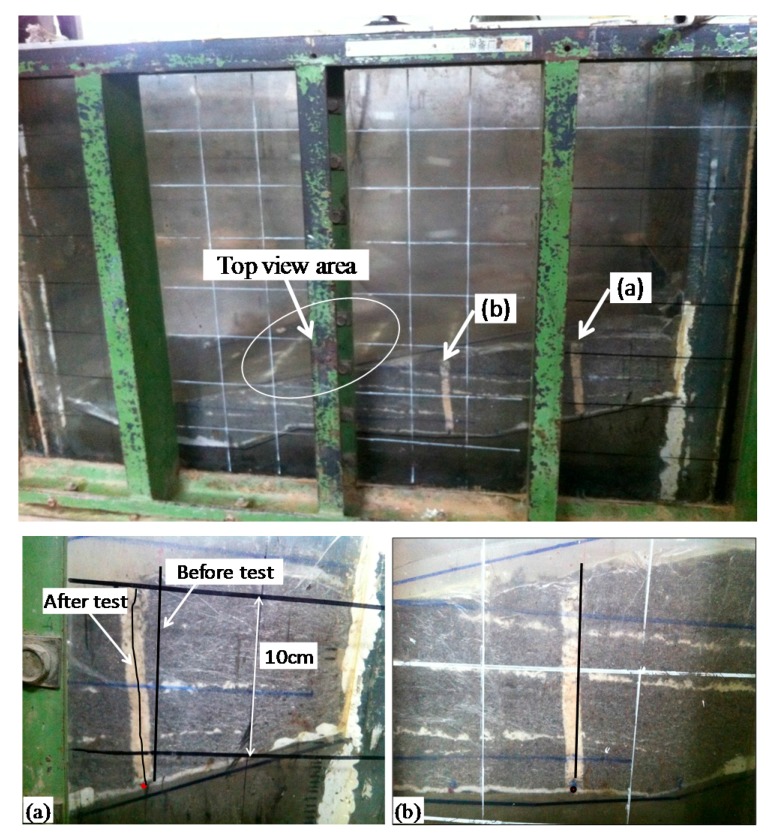
Photographs of failure mode (side view).

**Figure 18:**
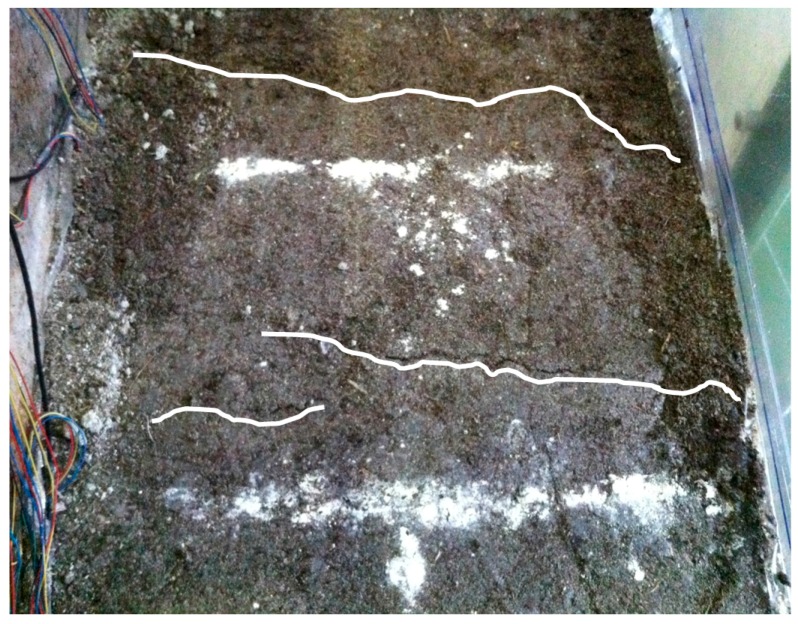
Photograph of failure mode (top view).

Figure 17 and Figure 18 show the photographs of the model slope failure. The slope did not experience a large slide but tension cracks were developed in the landfill indicating the onset of sliding.

Deep slip surface along the interface of geotextile and geomembrane in the liner system at the side slope was observed. The slip distance at this location of the model was up to about 1 cm, but at the base of the slope, the displacement between the GT/GM interfaces was smaller, as shown in Figure 17. It means that the shear stress on the liner was less than the shear strength. The results show that slippage first occurred along the side slopes of the landfill, and the failure continued to develop along the base towards the toe of the landfill. This failure mode is consistent with Filz *et al.*’s [20] finite difference analyses, where the progressive failure mechanism has been used to explain this phenomenon.

In addition, as can be seen in Figure 14, the model waste has experienced a larger auto-settlement before the failure of the landfill slope. So, for this failure mode, it is a challenge to select parameters for the slope stability calculation. It is because the MSW is a strain hardening material [11], but geosynthetics interface possess mainly strain softening properties. Based on the numerical analysis in Section 4, it is necessary to develop a property constitutive model which can reflect the characteristics of the MSW and the geosynthetics interface. Then, both the stability and lining system integrity could be considered.

It is important to note that the section in Figure 7 is much closer to the bulge area, where a ridge extended to the bottom of landfill site. Due to the steep terrain, a deep sliding deformation might appear at this location. But when considering the overall stability of the slope, x–y section that used in this model test (as shown in Figure 2) is a more representative area that is closer to the middle portion of the sliding mass. Hence, there are slightly different deformation and failure characteristics between the centrifugal model test and the monitoring results. On the other hand, the data collected under controlled laboratory conditions are useful for verifying the results of the analytical and numerical modelling methods.

## 6. Conclusions 

Based on the monitored field data at Shenzhen landfill and the back-analyses, the following conclusions are reached:

(1) Due to the failure of the leachate drainage system, the water level was relatively high. With increasing infiltration, this led to a further rise in the water level, which increased the weight of the MSW. As such, the waste body experienced a large deformation, which triggered the landslide within the liner system under the drag load.
(2) The rescue measures implemented after the landslide proved to be effective. These measures included pumping and draining water in the waste body so as to lower the water level; laying plastic film on top of the waste body so as to reduce infiltration to the body; unclogging the drains so that the surface water can flow out; and using sandbags to exert a back pressure to the bulging area at the foot of the slope.
(3) Based on interface test results of the geosynthetics, the shearing behavior of the landfill composite liner system was investigated using FLAC numerical simulation to conduct the integrated shearing test. The output data include the axial strain in geosynthetics, relative shear displacements and deformations. It provides a basis for decision-making, which used a simple geosynthetic liner system in centrifugal model test.
(4) For a typical geological profile of the landslide, a centrifuge model was used to quantitatively evaluate the effect of water level on the landfill instability. The failure process shows that progressive failure can occur along geosynthetic interfaces in lined waste landfills when peak strengths are greater than residual strengths.

This research provides an improved understanding of the physical behavior and failure mode of MSW landfill subjected to high water level. It has also shown that the deformation characteristics of waste (strain hardening), and the strain softening properties of geosynthetic interfaces should be considered in future stability analysis.
